# Initiation of Human Immunodeficiency Virus Type 1 (HIV-1) Transcription is Inhibited by Noncytolytic CD8^+^ Suppression

**DOI:** 10.2174/1874357900701010001

**Published:** 2007-08-13

**Authors:** R Glenn Overman, Anthony L Llorens, Michael L Greenberg, Mariano A Garcia-Blanco, Georgia D Tomaras

**Affiliations:** 1 Department of Surgery, Duke University Medical Center, Durham, NC, USA; 2 Department of Immunology, Duke University Medical Center, Durham, NC, USA; 3 Department of Medicine, Duke University Medical Center, Durham, NC, USA; 4 Department of Molecular Genetics and Microbiology, Duke University Medical Center, Durham, NC, USA; 6 Trimeris, Inc., Morrisville, NC, USA

## Abstract

The replication of human immunodeficiency virus type 1 (HIV-1) can be inhibited by noncytolytic CD8^+^ T cell mediated suppression, an immune response that specifically targets HIV-1 gene expression. Clinical studies demonstrate that this immune response may play an important role in the host defense against HIV infection. In this study, we examined the distinct steps in viral gene expression for inhibition by noncytolytic CD8^+^ T cells. A primary HIV-1 infection system of CD4^+^ enriched peripheral blood mononuclear cells was utilized to examine the HIV-1 life cycle as a relevant *ex vivo* system. Established CD8^+^ T cell lines from two HIV^+^ long-term nonprogressors were used to examine differences at the level of transcriptional initiation and elongation of the HIV genome. This infection system coupled with the results from real-time measurement of newly transcribed RNA transcripts determined that there was a significant decrease (5-8 fold) in short intracellular viral RNA transcripts. These data strongly favor a role for the initiation of virus transcription in noncytolytic CD8^+^ T cell mediated suppression.

## INTRODUCTION

The concept of non-cytolytic CD8^+^ T cell mediated suppression of HIV-1 unfolded in a seminal study by Levy’s group [1] that showed that CD8^+^ T lymphocytes can inhibit HIV replication in a dose dependent manner. This activity does not require HLA histocompatibility [2], is distinct from the cytolytic activity of CD8^+^ cells [3, 4] and is mediated by both cell contact and soluble molecules [5, 6]. The potency of noncytolytic CD8^+^ T cell mediated activity from HIV infected individuals *in vitro* has been shown to inversely correlate with disease progression [7, 8]. Several studies have confirmed that the CD8^+^ T cell mediated activity positively correlates with a healthier clinical status [8-11]. In addition, a significant number of exposed uninfected individuals demonstrate noncytolytic CD8^+^ T cell mediated suppressive activity [12, 13]. These clinical studies indicate that CD8^+^ T cell mediated antiviral activity may play an important role in the host defense against HIV infection and thus have a great potential to be of use in vaccination strategies. These results will contribute to the goal of identifying the specific antiviral mechanisms, and viral and cellular components, that contribute to the control of viremia by noncytolytic CD8^+^ T cell suppression.

The precise mechanism of CD8^+^ T cell mediated noncytolytic activity has not yet been fully delineated. The inhibition of virus replication can potentially take place at many different steps in the virus life cycle and therefore there is an array of molecules that may be the target of suppressive activity. It is known that for the inhibition of viral entry of CCR5-tropic viruses, the β-chemokines, MIP-1 α, MIP-1 β, and RANTES, can inhibit HIV replication by competitive inhibition of virus-coreceptor binding and downregulation of coreceptor cell surface expression [14-16]. The activities of these β-chemokines appear to act at entry, and not HIV-1 gene expression [17]. In addition, several well-characterized molecules have been tested for their ability to recapitulate this activity, but none can fully account for either the potency of suppression or the breadth across CXCR4 and CCR5-tropic viral infections [6].

In studies to examine the ability of CD8^+^ T lymphocytes to act on viral gene expression, primary CD4^+^ T lymphocytes were transfected with LTR driven reporter constructs to measure the effects of CD8^+^ T cells on both basal and Tat mediated transcription of the HIV-1 LTR [18]. CD8^+^ T cells from asymptomatic HIV-1 infected individuals could inhibit both basal and Tat mediated transcription and other work further substantiated these findings [19, 20]. Importantly, these affects on viral gene expression were also observed and confirmed using a primary lymphocyte culture in an ongoing cycle of viral replication where potent suppression of CXCR4-tropic viruses was shown not to involve entry or reverse transcription [21]. In addition, noncytolytic CD8^+^ T cell mediated suppression does not target viral integration [22]. These studies, utilizing both reporter constructs and ongoing cycles of virus replication in primary lymphocytes, indicate that CD8^+^ T lymphocyte suppression occurs sometime between proviral integration and splicing. However, it was not clear at what stage of virus transcription the antiviral activity was targeting. Transcription of the viral genome involves a complex series of events that trigger the initiation of transcription by the assembly of factors at the viral promoter. Once the transcript is initiated, another series of molecular interactions between the viral RNA and RNA binding proteins trigger elongation of the viral transcript. Previous studies examining the mechanism of action of noncytolytic CD8^+^ T cells did not differentiate between initiation and elongation of virus transcription. Utilizing an approach that distinguishes the molecular events occurring during HIV transcription, we demonstrate that noncytolytic CD8^+^ T cell mediated suppression targets initiation of virus transcription, but does not significantly affect virus transcript elongation. In addition to providing a better understanding of the mechanism of CD8^+^ antiviral activity, the results presented here could lead to the design of novel therapeutics that specifically inhibit the initiation of viral RNA transcripts.

## MATERIALS AND METHODS

### Isolation and Preparation of Primary CD8^+^ T lymphocytes.

Peripheral blood mononuclear cells (PBMCs) were prepared from venous blood from a seronegative donor (nEW, HIV-1 Naïve CD8^+^) by standard Ficoll-Hypaque density separation. PBMCs were activated for 3 days with anti-CD3 (OKT3) and anti-CD28 antibodies at 37°C in a humidified CO_2_ incubator. CD8^+^ T cells were isolated by negative selection with anti-CD4^+^ antibody coated beads (Dynal, Lake Success, NY). CD8^+^ cells from two asymptomatic long-term nonprogressors (LTNP) (JRHVS CD8^+^ and HS HVS CD8^+^) displaying potent suppressive activity for both X4 and R5 viruses were transformed with *Herpesviru*s *saimiri* (HVS) subgroup C strain 488-77 as previously described [24] and characterized [21]. IRB approval was obtained for all work with human specimens.

### Pseudotyped Virus Production.

 Pseudotyped viruses were produced by transfection of DNA into the human embryonic kidney cell line, 293T, using FuGENE (Roche Inc.). The reporter virus DNA, pNL4-3.LUC.R^-^E^-^ [obtained through the NIH AIDS Research and Reference Reagent Program from Dr. Nathaniel Landau [25, 26] was cotransfected with the expression vector pNL4-3 *env* (a gift from Dr. T. Dragic). The virus-containing supernatants were harvested two days later, filtered through a 0.45µm membrane and stored at -80° C in 20% v/v FBS. Viral tropism was tested in a human astroglioma cell line expressing either CD4^+^ alone (U87.CD4), CD4^+^ and CCR5 (U87.CD4.CCR5), or CD4^+^ and CXCR4 (U87.CD4.CXCR4) all obtained through the NIH AIDS Research and Reference Reagent Program from Drs. HongKui Deng and Dan Littman [27]. U87 cells were grown in DMEM L-glutamine containing 1% Pen/Strep, 15% FBS, and selective agents (300 µg/ml G418 for CD4 alone cell lines, or 300 µg/ml G418 plus 1 µg/ml puromycin). In triplicate, U87 cells were seeded in a 96-well flat bottom plate at 7.5 × 10^3^ cells/well and grown overnight at 37°C. The selective media was then removed and 100 ul of pseudotyped virus was added. Cells were infected for 2.5 hours at 37°C. 100 ul of fresh media without selective agents was then added.

To assess viral tropism, at 72 hours post-infection the media was removed and cells gently washed with warm PBS. U87 cells were then lysed with 50 µl of 1X Cell Culture Lysis Reagent (Promega, Madison, WI) and then frozen for >30 min at -80°C. 20 ul of each lysate was assayed with the Promega Luciferase Assay System on an EG&G (Berthhold) LB 96V luminometer and viral infection was measured in relative light units (RLU).

### CD8^+^ Suppression Assay.

Suppression assays were performed as previously described [21] with modifications as indicated. Peripheral blood mononuclear cells were prepared from freshly drawn blood from a pool of HIV^-^ donors and activated as described above. CD4^+^ and CD8^+^ enriched populations were obtained by negative selection with anti-CD8^+^ or anti-CD4^+^ antibody coated beads (Dynal, Lake Success, NY), respectively. CD4^+^-enriched PBMC were infected with 1 ml of pseudotyped virus per 1 × 10^6^ CD4^+^ cells in the presence of DEAE (20 µg/ml) for 2.5 hours at 37°C. The cells were washed with PBS and resuspended at 4.5 × 10^5^cells per ml in AIM-V medium (Invitrogen, Carlsbad, CA) containing 20% v/v FBS and 40 U/ml IL-2. Previously enriched effector CD8^+^ cells from LTNP and HIV^-^ donors were added at a 2:1 E:T ratio achieving a final concentration of 1.35 × 10^6^ cells/ml. 200 µl aliquots of the infectious cultures, in triplicate, were transferred to a 96-well flat bottom microtiter plate and incubated at 37°C for 48 hrs.

At 48 hours post-infection, the cells used for RNA isolation were pelleted and resuspended in a 1:5 v/v PBS:RNAlater (Ambion, Austin TX) solution, stored at 4°C overnight, then transferred to -20°C. To assess infection, cultures in the microtiter plate were washed with PBS at 48 hours, lysed with 1X Cell Culture Lysis Reagent (Promega, Madison, WI) and then frozen for >30 min at -80°C. 20 ul of each lysate was assayed with the Promega Luciferase Assay System on an EG&G (Berthhold) LB 96V luminometer. Luciferase activity was measured in relative light units (RLU).

### Viral RNA Analysis.

Total RNA, including low molecular weight RNA, was isolated from the RNAlater-stored cell samples using the *mir*Vana miRNA Isolation Kit (Ambion) according to manufacturer’s instruction. DNA was removed with a double DNase digest treatment using DNA-free (Ambion). The RNA samples were quantified using a spectrophotometer and then electrophoresed on a 1% agarose gel to check the integrity. Real-time PCR with HIV specific primers (HIVshort-F and HIVshort-R) and host RPS9 specific primers (RPS9-F and RPS9-R1) was then performed to check for DNA contamination against a standard curve, described below. The DNA-free RNA was then reverse-transcribed (Thermoscript, Invitrogen) using reverse primers (Table **1**) at 119 bp, ∼2.9 kb, ∼7.1 kb, and a luciferase region (9.7 kb) of the pNL4-3.LUC.R^-^E^-^ genome (GenBank Accession number AF324493.1).

Host gene RPS9 primer locations were at 418 and 5579 bp (Ensembl Gene ID ENSG00000170889) (Tables **1**,**2**). Transcript copy numbers for the above-mentioned locations were determined by real-time PCR on an ABI 7500 (Applied Biosystems, Foster City, CA) using SYBR Green PCR Master Mix (Applied Biosystems). A melting curve analysis was performed on each run to insure amplicon specificity. A standard curve (plasmid copy range 10^2^- 3 × 10^6^) was generated using plasmid pNL4-3.LUC.R^-^E^-^ with primers specific to the 119 bp region of interest. Acceptable slopes (M) for standard curves ranged from -3.1 to -3.6 (optimal slope, M= -3.3). All best fit lines demonstrated R^2^ values > 0.98. Note: all primers were designed to yield amplicons of analogous size, approximately 100 bp, and were chosen for similar amplification efficiencies. The RNA extracted from each viral infection experiment was reverse transcribed in duplicate, and cDNA from each of the duplicates was used in two separate real-time PCR experiments. The cDNA templates used in the real-time experiments were assayed in triplicate.

## RESULTS

### Noncytolytic CD8^+^ T Cell Mediated Suppression System in CD4^+^ T Lymphocytes.

To examine the mechanism of noncytolytic CD8^+^ T cell mediated suppression during the time of viral gene expression, we utilized a single cycle infection assay in primary CD4^+^ lymphocytes. Two established cell lines (JR-HVS CD8^+^ and HS-HVS CD8^+^) that mediate suppressive activity [21] were utilized to determine if viral RNA transcripts are inhibited at transcriptional initiation and/or elongation. In a manner analogous to the non-transformed cells from which they were derived, the HVS-transformed CD8 cells were shown to potently inhibit infection of both X4- and R5- dependent viruses [21]. Here we utilize the CD8^+^ effectors and CD4^+^ targets that were previously determined by our group to lack any demonstrable cytolytic [21] and allogeneic reactivities. This system allows the isolated study of the effects of noncytolytic CD8^+^ suppression on HIV replication in a system where we have previously measured the kinetics of HIV entry, reverse transcription and early gene expression [21]. Fig. (**1**) shows the results of CD8^+^ suppression assays using a CXCR4-tropic enveloped (NL4-3) pseudotyped virus. The CD8^+^ effectors (JR HVS CD8^+^ and HS HVS CD8^+^) demonstrated a greater than one-log reduction in viral infection in contrast to the HIV-1 naïve CD8^+^ cells (nEW CD8^+^). We did note that there was more variation in the level of infection in the presence of the nEW CD8^+^ cells, however the CD8^+^ cells derived from the HIV^+^ LTNP significantly suppressed HIV replication compared to nEW CD8^+^ cells.

### Noncytolytic CD8^+^ T Cell Mediated Suppression of HIV Gene Expression.

 To investigate the possible mechanisms of suppression at transcriptional initiation and elongation, we utilized a system that combines reverse transcription of viral RNA transcripts with quantitative real time PCR (adapted from [28]). RNA was isolated from the CD8^+^ suppression assays shown in Fig. (**1**). Reverse transcription was utilized to construct cDNAs, and then real-time PCR was used to quantify viral RNA transcripts at various points in the HIV genome. Fig. (**2**) shows the different regions of the HIV genome that were reverse transcribed and then quantified using real-time PCR. HIV RNA transcript copy numbers were measured by quantitative real-time PCR at the following positions along the HIV genome: 119 bp (HIVshort-R and HIVshort-F primers), 2,958 bp (3 kb-R and 3 kb-F), 7,120 bp (7 kb-R and 7 kb-F), and 9,700 bp (Luc-R and Luc-F). All short and longer transcripts were reversed transcribed from the RNA template using primer sets that resulted in amplicons of similar size, “short amplicon”, to accurately quantify and compare both long and short transcripts. Fig. (**2**) diagrams the RNA transcripts and PCR amplicons for both the short transcript that tests transcriptional initiation and also for one of the longer elongated transcripts (3 kb) as an example. Primers specific to a housekeeping gene, ribosomal protein (RPS9), were utilized at comparable positions on the gene as a control.

### Viral Suppression by CD8^+^ Cells Occurs at Initiation.

**Fig. (3):**
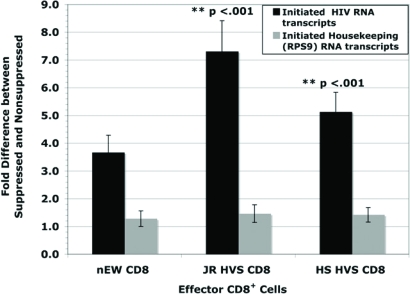
Reduction in Initiation of Transcription during Noncytolytic CD8^+^ T cell mediated Suppression of HIV Replication. Both JR-HVS and HS-HVS significantly suppressed viral transcription initiation more than the HIV naїve CD8^+^ T cells (nEW CD8^+^), p < 0.001 for both comparisons, Student t-Test. The fold difference between the suppressed and nonsuppressed viral cultures were determined by quantifying cDNA copy numbers from the short RNA transcripts using real time PCR.

To define whether or not suppression occurred at transcriptional initiation the *initiation ratio* of non-suppressed HIV short copy number to suppressed HIV short copy number [NSS/SS] was examined. The use of a single-cycle infection prevented subsequent rounds of virus replication and allowed the observance of a single viral replication cycle. At 48 hours post infection, the initiation ratio demonstrated that the JR HVS CD8^+^ and HS HVS CD8^+^ cells executed significant suppression at the initiation stage of viral transcription, on average 7.3 fold and 5.1 fold inhibition respectively (Fig. **3**). The HIV-naïve CD8^+^ cells also exhibited inhibition, 3.7 fold on average, albeit not to the strength of LTNP CD8^+^ cells. Interestingly, this lower level of inhibition of initiation was consistent regardless of the level of infection in the presence of HIV-naïve CD8^+^ cells. Both the JR HVS CD8^+^ and HS HVS CD8^+^ cells suppressed transcriptional initiation to a significantly higher level than the HIV-naïve CD8^+^ cells (p < 0.001 for both comparisons, Student’s t-test).

The housekeeping gene, ribosomal protein S9 (RPS9), was measured to determine if the CD8^+^ cells had a general effect on suppressing host cell transcription. From the same RNA isolates for every experiment, real-time PCR was used to quantify early transcription of the housekeeping gene. The data as shown in Fig. (**3**) illustrates there was no suppression of the housekeeping gene, RPS9 supporting the finding that CD8^+^ T cell mediated suppression is unique to HIV transcription.

### Measurement of Transcripts that have completed Elongation and Splicing.

 Primers specific to near the 3 kb site of the HIV genome were designed to address the question of whether noncytolytic CD8^+^ T cell mediated suppression affects transcriptional elongation. An equation NSL/[SL x [NSS/SS]] was derived so that any contributing factors from transcriptional initiation could be included. Fig. (**4**) shows data from HIV short and 3 kb primers with the resulted values of JR = 0.6, HS = 0.6, and EW = 0.8. The lack of significant effect (controlling for the suppressive effects at viral initiation) at approximately 3 kb from the start of viral transcription indicates that suppression of virus replication is likely limited to the initiation of transcription. In addition, RT-PCR was also used to quantify the late transcription products of the housekeeping gene, RPS9. There was no significant effect on RPS9 elongation. Because both RPS9 transcriptional initiation and elongation are both near a 1:1 ratio between non-suppressed and suppressed systems, this supports that there is no global effect of CD8^+^ inhibition on general host cell gene expression. This supports previous data that non-cytolytic CD8^+^ suppression is specific to HIV replication [18].

The analysis of viral transcript elongation (including splicing) was extended further along the HIV genome to test for possible amplification of smaller effects downstream from those potentially present at shorter distances from the start of virus transcription. Fig. (**5**) shows the viral cDNA copy number measured at 3 kb, 7 kb, and 9.7 kb from the start of transcription for each of the four experimental conditions (from the same set of samples examined in Fig. (**4**)). The fold difference between the suppressed and non-suppressed viral cultures were determined by quantifying cDNA copy numbers using real-time PCR and then controlling for differences observed at transcriptional initiation. As was expected, the HIV short transcript (119 bp) was the most prominent in copy number in both non-suppressed (positive control) and suppressed systems. Based on the ratio NSS/NSL [non-suppressed short/ non-suppressed long viral RNA transcripts], which measures the progression from initiation [HIV short] to elongation (HIV long-3 kb), on average there are 3.8 times as many HIV short transcripts than 3 kb transcripts in positive controls (N = 16). The addition of JR HVS CD8^+^ and HS HVS CD8^+^ effector cells to infected CD4^+^ T lymphocytes resulted in greater HIV short to 3 kb ratios, however, this was to a lesser degree, 2.9 and 2.1, respectively (N = 16 and N = 12). There were fewer 3 kb copies than 7 kb or 9.7 kb copies in all samples tested and this was interpreted to be a consequence of the alternative splicing of HIV-1 transcripts.

Comparing the ratios of the HIV short transcript to either the 7 kb transcript or the 9.7 kb transcript, there was no difference between ratios for the positive controls and the CD8^+^ T cell effectors. These data provide evidence that the inhibition of viral replication by noncytolytic CD8^+^ suppression did not likely involve transcriptional elongation or splicing, but occurred predominately at the initiation of viral transcription.

## DISCUSSION

Here, it is reported that noncytolytic CD8^+^ suppression of HIV replication is specific to the initiation phase of viral transcription. Previous studies demonstrated the involvement of HIV transcription [18, 19], but did not measure the distinct molecular steps of transcription: initiation and elongation. Expression of HIV-1 genes and inhibition by noncytolytic CD8^+^ suppression begins to occur by 17-25 hours after infection [21]. By choosing to evaluate suppression at the 48-hour time point, basal and/or tat-mediated transcription occurred and were possible targets, while CD8^+^ suppression was still in an optimal phase.

HIV-1 transcription is regulated by a complex interaction of cellular and viral proteins. Sequences within the HIV-1 genome that are involved in viral gene transcription can be divided into the core promoter elements, the enhancer, the modulatory and negative regulatory elements and the tat responsive element (TAR). HIV transcription is primarily regulated by the viral promoter (5' long terminal repeat) which contains a canonical RNA polymerase II (RNAPII) TATA box, transcription factor binding sites and an initiator element [29]. Subsequent to the initiation of transcription, elongation occurs by competent RNAPII complexes. During transcriptional activation, the viral transactivator, Tat [30], binds to a viral RNA structure, the *trans*-activation response element (TAR) to increase the level of transcription by greater than 2-logs [31, 32]. Positive transcription elongation factor b (P-TEFb), Tat and TAR form a trimeric complex that *via* one of P-TEFb’s components, CDK9 (a CTD kinase), phosphorylates the C-terminal heptapeptide repeate domain (CTD), of the largest subunit of RNAPII. Phosphorylation of CTD is thought to mediate the increase in transcript elongation.

Our work suggests that anti-viral CD8 T cells may suppress the transition from the initiation of viral transcripts to elongation of these same transcripts. The mechanisms and factors that could be involved include the alteration of CTD phosphorylation sites or modulation of Tat activities. Tat is primarily thought to be involved in transcriptional elongation, but several labs have presented evidence that Tat is likely to be involved in initiation by aiding in the assembly of the transcription complex (TC) (reviewed in [33]). Understanding whether anti-viral CD8^+^ T cells act to modulate Tat activity to inhibit HIV replication will be important to consider as potential pathways for the inhibition of viral transcriptional initiation are explored.

Although the data generated here do not support CD8^+^ suppressive activity at elongation and/or splicing, it is important to note that this possibility cannot be fully eliminated. The role of C-terminal heptapeptide repeat domain (CTD) of RNAP II, CA150 and/or the Tat-P-TEFb complex (which includes cyclin T1) has yet to be fully investigated for their potential roles in inhibition of HIV.

There is some controversy as to the ability of HIV-naïve CD8^+^ cells to suppress HIV infection. It is reported here that HIV-naïve CD8^+^ cells inhibit transcriptional initiation of HIV, but the magnitude is less than that of the CD8^+^ T lymphocytes derived from long-term nonprogressors. This is in agreement with the lower level of suppression of virus infection by these HIV-naïve CD8^+^ T cells observed here. We can not exclude the possibility that the transformation of the CD8^+^ lymphocytes may have contributed to the transcriptional effects demonstrated here, although we consider it unlikely as previous work by our group demonstrated that the transformed cells recapitulated the phenotype of the primary cells from which they were derived [24].

In the system presented here both soluble and contact mediated non-cytolytic CD8^+^ cell suppression of viral replication are permissible. Previous studies have shown that CD8^+^ T lymphocytes express and secrete soluble molecules capable of suppressing viral replication. The most notable of these molecules are the natural ligands for the CCR5 coreceptors, the β-chemokines RANTES, MIP-1α and MIP-1β, which do in fact inhibit viral replication by blocking viral entry. Since their discovery, however, several groups have shown that these soluble anti-viral factors cannot adequately explain the viral suppressive activity of CD8^+^ T cells [34, 35]. Here we report that the anti-viral suppression occurs during transcriptional initiation of the HIV viral life cycle (using X4-tropic virus) and this will further ongoing studies into the precise molecules involved in suppressing viral transcriptional initiation.

Other than HIV-1, Hepatitis B virus is another virus described as sensitive to non-cytolytic CD8^+^ cell suppression activity [36]. The use of *tat*-mediated enhancement of viral transcription is a characteristic that differentiates HIV transcription from Hepatitis B transcription. HIV and Hepatitis B are both inhibited by non-cytolytic CD8^+^ T lymphocyte suppression as both also use host cell transcriptional machinery for transcription of viral RNA. Considering that HIV utilizes a unique means of enhancing transcription through the HIV *tat*-protein and TAR-sequence (tat-activating region), and both HIV and Hepatitis B virus use host cell transcription machinery, non-cytolytic CD8^+^ suppression could be mediated by altering host cell transcription machinery that may act specifically in concert to target viral transcription. This idea is further supported by the complexity of cellular transcription and the numerous possible targets for modification by host cell defense mechanisms.

As provided in this report, the use of an *ex vivo* system provides strong data showing that CD8^+^ T cell suppression occurs at viral transcription. Future studies will focus on *in vitro* transcription and protein expression analysis to identify the molecular players. This discovery may provide some insight into how CD8^+^ T cells from long-term nonprogressors and exposed uninfected individuals may suppress HIV replication. Further uncovering of the exact mechanism around the initiation of viral transcription may lead to the development of novel therapeutics for HIV infection or innovative means to elicit this CD8^+^ activity as part of a vaccine strategy.

## Figures and Tables

**Fig. (1):**
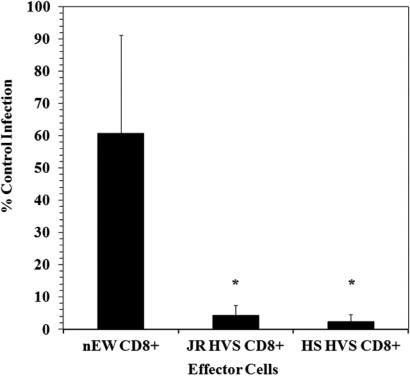
Noncytolytic CD8^+^ T Cell Mediated Suppression of HIV NL4-3 Pseudotyped Virus Replication. CD4^+^ T lymphocytes were infected with NL4-3 pseudotyped virus and exposed to CD8^+^ T lymphocytes. (N=4) % Control Infection is the amount of infection relative to the control NL4-3 infected CD4^+^ T-lymphoctyes. The cutoff for the upper limit for suppression was 10% infection, a 1-log reduction, in the CD4^+^ co-cultures with JR HVS CD8^+^ and HS HVS CD8^+^. ^*^ p = 0.023 for both JR and HS HVS CD8^+^ compared to nEW CD8^+^, Student t-Test.

**Fig. (2):**
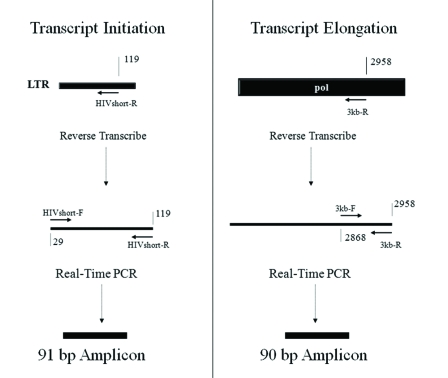
Quantification of HIV RNA Transcripts from the HIV-1 Genome during Cellular Infection. Transcriptional initiation and elongation were examined using quantitative RT-PCR that measures RNA transcripts that reach a relatively short distance and also a much larger distance from the start of transcription. Reverse transcription was performed using primers near the LTR (“HIV short-R”) and within the pol gene (“3kb-R”). The short and long transcripts were quantified using primer sets of equivalent efficiency that amplified transcripts of similar length (“HIVshort-F and HIVshort-R” for the short cDNA transcript and “3kb-F and 3kb-R” for the long cDNA transcript). Strategy adapted from Sune *et al.* [28].

**Fig. (4):**
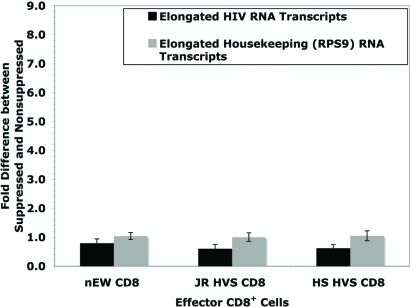
Transcriptional Elongation/Splicing during Noncytolytic CD8 T cell mediated Suppression of HIV Replication. Using the derived equation [NSL/[SL x [NSS/SS]]], this figure illustrates the calculated elongation/splicing effects from non-cytolytic CD8^+^ suppression as determined by the “fold difference between suppressed and non-suppressed” viral cultures. By accounting for inhibition at transcriptional initiation, ratio values of JR = 0.6, HS = 0.6, EW = 0.8 indicate the likely absence of suppression at elongation and/or splicing (N = 16, N = 12, and N = 16, respectively).

**Fig. (5):**
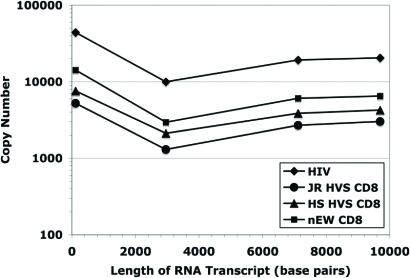
No significant Effects Past Initiation Regardless of Length of Viral RNA Transcript. The viral cDNA copy number was measured at four distances from the start of transcription for each of the four experimental conditions. When comparing copy number ratios, there is no significant difference (Student t-Test, p=0.331 to p=0.476) when comparing HIV short: 7 kb and HIV short: 9.7 kb transcript ratios in the positive controls *vs* suppressed systems (JR HVS CD8^+^, HS HVS CD8^+^) and nEW CD8^+^. Regardless of the length of the transcript examined, there are no significant effects downstream of initiation.

**Table 1.** 

Primer	Sequence [5’to 3’]	5’ Position [bp]
HIVshortF	CCTGGGAGCTCTCTGGCTAA	29
HIVshortR	AACAGACGGGCACACACTACTTT	119
3kb-F	CAATGACATACAGAAATTAGTGGGAAA	2868
3kb-R	CTTTGGTTCCCCTAAGAAGTTTACAT	2958
7kb-F	TGGCAGGAAGTAGGAAAAGCA	7040
7kb-R	AGCAGCCCAGTAATATTTGATGAAC	7120
Luc-F	AGGTGGCTCCCGCTGAA	*9.6 kb
Luc-R	ACACCTGCGTCGAAGATGTTG	*9.7 kb
RPS9-F	GACGGGGAAGCGGAGCCAACA	**308
RPS9-R1	GCTTCAGCTCTTGGTCGAGACGAG	**418
RPS9-R2	GCGAGCGTGGTGGATGGACTTG	**5579

* Primer locations are based on the luciferase gene sequence in Promega’s pGL-3 basic vector, forward primer at 1343 bp and reverse primer 1406 bp. The luciferase reporter gene is located in the *nef* gene of the HIV pNL4-3.LUC.R^-^E^-^ genome at 8.3 kb.

** Primer locations are based on Ensembl Gene ID: ENSG00000170889.

**Table 2:** 

Primer	Position mRNA NM_001013	Position Genome ENSG00000170889
RPS9-F	37	308
RPS9-R1	147	418
RPS9-R2	437	5579

The RPS9 primers were designed based on GenBank Accession number U14971.1. The primer positions are listed in reference to the 5’ base relative to ^+^1 transcription/gene start.
